# Risk Factors Associated with Failure and Technical Complications of Implant-Supported Single Crowns: A Retrospective Study

**DOI:** 10.3390/medicina59091603

**Published:** 2023-09-05

**Authors:** Adam Larsson, Justice Manuh, Bruno Ramos Chrcanovic

**Affiliations:** 1 Faculty of Odontology, Malmö University, 214 21 Malmö, Sweden; adamlarssonn00@gmail.com (A.L.); agyemangjustice8@gmail.com (J.M.); 2 Department of Prosthodontics, Faculty of Odontology, Malmö University, 214 21 Malmö, Sweden

**Keywords:** dental implant, fixed prosthesis, implant-supported single crown, technical complications, survival

## Abstract

*Background and Objectives*: Implant-supported single crowns have become a routine approach for the replacement of missing single teeth, being considered as one of the most common ways of rehabilitation when adjacent teeth are healthy. The present retrospective study aimed to investigate the risk factors possibly associated with failure and technical complications of implant-supported single crowns and their supporting implants. *Materials and Methods*: Patients treated at one faculty (2009–2019) were considered for inclusion. Complications investigated included ceramic fracture/chipping, crown loss of retention/mobility, crown failure/fracture, loosening/loss/fracture of prosthetic screw, and implant failure/fracture. Any condition/situation that led to the removal/replacement of crowns (implant failure not included) was considered prosthesis failure. Univariate/multivariate Cox regression models were used to evaluate the associations between clinical covariates and failure. *Results*: 278 patients (358 crowns) were included. Mean ± SD follow-up was 56.5 ± 29.7 months. Seven implants (after a mean of 76.5 ± 43.7 months) and twenty crowns (21.3 ± 23.5 months) failed. The cumulative survival rate (CSR) for crowns was 93.5% after 5, remaining at 92.2% between 6 and 11 years. The most common reasons for crown failure were porcelain large fracture (*n* = 6), crown repeatedly loose (*n* = 6), and porcelain chipping (*n* = 5). Men and probable bruxism were identified in the Cox regression model as being associated with crown failure. The most common observed technical complications were mobility of the crown and chipping of the ceramic material, with the latter being observed even in crowns manufactured of monolithic zirconia. Cases with at least one technical complication (not considering loss of screw hole sealing) were more common among probable bruxers than in non-bruxers (*p* = 0.002). Cases of ceramic chipping were more common among bruxers than in non-bruxers (*p* = 0.014, log-rank test). *Conclusions*: Probable bruxism and patient’s sex (men) were factors associated with a higher risk of failure of implant-supported single crowns.

## 1. Introduction

The first well-established protocol for oral rehabilitation with modern dental implants advocated the placement of multiple implants in the totally edentulous jaws to support full-arch fixed dental prostheses [1]. The use of modern implants developed until reaching the stage of being used for the replacement of the loss of single teeth. Schmitt and Zarb [2] were probably the first ones who reported the clinical outcomes of a series of implants used for the prosthetic replacement of single teeth.

Implant-supported single crowns have become a routine approach for the replacement of missing single teeth, being considered as one of the most common ways of rehabilitation when adjacent teeth are healthy, and/or when the patient refuses the dental hard tissue reduction by preparation with drill bits in order to fit a three-unit fixed partial denture supported by natural tooth abutments [3,4].

Implant-supported single crowns have many other advantages over short-span tooth-supported fixed partial dentures besides the fact that teeth are not prepared. Adjacent teeth would have a better prognosis with a decreased risk of abutment tooth loss, as they are not subject to a higher incidence of endodontic therapy and decay as a result of tooth preparation, and the patient would have an improved ability to clean the proximal surfaces of the adjacent teeth [4,5]. The outcome with the implant single crown is usually regarded as economically superior compared to the tooth-supported prosthesis [6], maintenance of bone in the edentulous site, and psychological advantage [7]. Yet, single crowns supported by dental implants are not free from technical complications and failure [8]. Complications such as either loosening or fracture of the prosthetic screw may happen, consequently resulting in mobility or even complete looseness of the prosthetic crown. Decementation of the crown may occur in cases where the crown is cemented to a prosthetic abutment instead of being screwed directly to the implant. Chipping or fracture of the crown restorative ceramic material may take place, resulting in deficits of the esthetic appearance, which may require chair-side intervention (such as in-mouth polishing of the chipping) or the prosthesis to be sent to the dental lab for reparation, or even replacing the prosthetic crown entirely by a new manufactured one. Moreover, fracture or deformation of the prosthetic abutment may happen [8].

The survival rates of implant-supported single crowns recently reported in the literature usually are high, with a rate variation depending mostly on the extension of the follow-up and on the definition of what failure would stand for in a particular study. According to a recent retrospective study that investigated more than 500 crowns with a mean follow-up of 15 years, the failure rate can vary between 4.1% and 9.5%, depending on whether non-technical complications that lead to crown replacement, such as esthetic issue, change to another type of prosthesis, crown in infraposition, or raise of the patient’s bite, are considered or not [8]. Other studies with smaller cohort groups and shorter follow-up periods reported higher survival rates, such as 97.5% after a mean of 61 months of follow-up of 40 implant-supported crowns [9], and 100% after a mean of 80.9 months of follow-up of 31 crowns [10]. A study with a similar cohort group size, with 482 implant-supported single crowns, reported a complication rate of 3.1%, but with a relatively short mean follow-up of 5 years [11].

The aim of the present retrospective clinical study was to investigate the risk factors that could be associated with the occurrence of failure and technical complications of implant-supported single crowns. The null hypothesis was that there would be no factor associated with the occurrence of failures of implant-supported single crowns, against the alternative hypothesis of some factor having some influence on crown failure.

## 2. Materials and Methods

### 2.1. Patients

This retrospective study included patients treated with implant-supported single crowns from March 2009 to December 2019 at the Faculty of Odontology, Malmö University, Malmö, Sweden. This study was based on data collection from patients’ dental records. The study was approved by the regional Ethical Committee, Lund, Sweden (Dnr 2018/856).

The cases were initially screened in the software (T4 Practice Management Software, Version 2, Carestream Dental LLC, Atlanta, GA, USA), by looking for cases with the help of the codes given for the treatment in focus.

### 2.2. Definitions

Implant failure: An implant was considered a failure if presenting signs and symptoms that led to implant removal, i.e., a lost implant. Implant failure could be either early (the inadequacy of the host to establish or promote osseointegration in the early stages of healing) or late (the failure of either the established osseointegration or function of dental implants) [12]. Implant fracture was also considered as a failure [13].

Prosthesis failure: Any condition or situation that led to the removal and/or replacement of the crown was considered as a prosthesis failure. Implant failure per se was not considered as a crown failure. Any other condition or finding was considered as a complication.

Technical and non-technical complications were defined according to a previous study [8].

Bruxism: The criteria for the diagnosis of ‘probable’ bruxism, used in a previous study [14], was adopted for the present study. Probable bruxism was based on self-report, by means of questionnaires and/or the anamnestic part of a clinical examination, together with the inspection part of a clinical examination [15]. The clinical signs of bruxism could include one or a combination of the following: masticatory muscle hypertrophy, indentations on the tongue or lip, linea alba on the inner cheek, mechanical wear of teeth, damage to the dental hard tissues (such as cracked teeth), and repetitive failures of restorative work and/or prosthodontic constructions [16]. Only patients with both self-report and clinical signs were considered probable bruxers.

Smoker: Patients smoking a minimum of one cigarette per day (an everyday smoker [17]) were classified as smokers.

### 2.3. Inclusion and Exclusion Criteria

All patients rehabilitated with one or more implant-supported single crowns during the aforementioned period were included. Only modern threaded cylindrical- or conical-design implants were considered for inclusion. Since it was observed, in a pre-analysis of the data, where some cases failed within the first month after prosthesis installation, it was decided that any case would be included based on the previous inclusion criteria, provided that some follow-up was recorded. Therefore, the cases not followed up at all, namely, patients who received the prosthetic restoration and never came back for an appointment, were excluded.

### 2.4. Sample Size Calculation

The calculation was based on the results of Chrcanovic et al. [8], which observed a prevalence of single crown failure of 9.5% over a mean follow-up period of over 15 years. As the mean follow-up for the present study was expected to be shorter, as patients treated between 2009 and 2019 were screened for inclusion, it was anticipated a half prevalence of prosthetic failure than observed in the aforementioned study, namely 4.75%. Therefore, there was a need for 252 cases in total, having set alpha (α) at 0.05 and power at 80%. The calculation was performed with ClinCalc.com (accessed on 29 May 2022).

### 2.5. Data Collection

The data collected consisted of the following: patient’s sex, patient’s age at the definitive crown delivery, smoking habits, presence or not of probable bruxism, presence of a systemic health issue, region of the crown according to the FDI numbering system, location of the implant in relation to jaw (maxilla or mandible), position of the tooth within the jaw (anterior comprising region of incisors and canine, posterior comprising region of premolars and molars), prosthetic crown material, crown fixation method (cemented or screw-retained), use of night guard, situation of the region of the opposing arch in contact with the single crown (natural teeth, fixed prosthesis-restored teeth, implant-supported fixed prosthesis, removable partial denture, removable total denture, overdenture, edentulous region), occurrence of technical complications, operator (dental student or graduated dentist), and follow-up time.

### 2.6. Statistical Analyses

Most of the methodology used for the statistical analyses has been described elsewhere [18] but applied for single crowns. Comparison of the frequency of ceramic chipping between bruxers and non-bruxers was performed by using the log-rank (Mantel–Cox) test, in a Kaplan–Meier analysis. For the prosthesis-level analysis, clustering of multiple single crowns within each patient was accounted for in the Cox regression models using the methods outlined by Lee et al. [19] and Lin [20].

## 3. Results

From March 2009 to December 2019, a total of 1193 implant-supported single crowns were installed at the Faculty of Odontology, according to the registry in the software. Many cases were excluded for not really being implant-supported single crowns; namely, the prosthetic restoration was a different one, but the code given for the treatment was mistakenly entered in the software as an implant-supported single crown. This was mostly true for implant-supported partial fixed prostheses when, for example, a two-unit prosthesis was given the code twice for an implant-supported single crown. At the end, 360 cases were considered for inclusion, of which 2 cases were excluded for not having any follow-up after a prosthetic single crown was installed. Therefore, 358 implant-supported single crowns in 278 patients fulfilled the inclusion criteria.

The mean age (±SD) of the 278 patients was 54.3 ± 12.6 years (min–max, 25.3–91.7) on the day of the crown installation. The patients were followed up for a mean (±SD) of 56.5 ± 29.7 months (min–max, 0.2–146.2) after prosthesis installation.

The mean (±SD) time between implant placement and prosthesis installation was 6.8 ± 2.8 months (min–max, 1.8–38.8). Most of the implants of the study were from Straumann (*n* = 177), followed by Astra (*n* = 121), Nobel Biocare (*n* = 42), Biomet (*n* = 15), and Southern, Camlog, and Ankylos (1 each). Seven implants failed, two Straumann and five Astra implants, after a mean (±SD) of 76.5 ± 43.7 months (min–max, 24.5–134.4). One of these failures was due to implant fracture (Astra Osseospeed, diameter 4.00, length 11.0, tooth region 46).

A number of 167 crowns (out of 358; 46.6%) were followed up for at least 5 years, and only 6 (1.7%) were followed up for at least 10 years. A total of 4 patients had four single crowns, 7 patients had three single crowns, 54 patients had two single crowns, and the remaining 213 patients had only 1 implant-supported single crown. Table 1 shows the descriptive data of the single crowns included in the study.

Table 2 presents the results of the life-table survival analysis. The cumulative survival rates (CSRs) were 93.5% and 92.2% for all single crowns after 5 and 6–11 years, respectively. Twenty crowns failed, after a mean follow-up (±SD) of 21.3 ± 23.5 months (min–max, 0.3–82.1). The reasons for failure were porcelain chipping in five cases, porcelain large fracture in six cases, crown repeatedly presenting episodes of looseness in six cases, crown in infraposition in one case, prosthesis misfit in one case, and crown overextended in 1 case.

Table 3 and Table 4 present the results for the univariate and respective multivariate Cox proportional hazard models. Multicollinearity was not detected. The hazard ratio (HR) was statistically significant for two factors in the univariate models (men and probable bruxism), and both remained statistically significant in the multivariate model. The difference in the distribution of bruxers between crowns placed in men (37/145) and in women (64/213) was not statistically significant (*p* = 0.350, Pearson’s chi-squared test).

The occurrence and frequency of technical complications are described in Table 5. The most common observed complications were mobility of the crown and chipping of the ceramic material. Occurrence of ceramic chipping was more prevalent among probable bruxers (20/101) than in non-bruxers (22/257). The difference between groups was statistically significant (*p* = 0.014, Kaplan–Meier analysis, log-rank test). Cases with at least one technical complication (not considering loss of screw hole sealing) were more common among probable bruxers (36/101) than in non-bruxers (52/257; *p* = 0.002; Pearson’s chi-squared test).

## 4. Discussion

The present retrospective clinical study aimed to investigate the risk factors that could be associated with the occurrence of failure and technical complications of implant-supported single crowns. According to the Cox proportional hazard models, probable bruxism and men were associated with prosthesis failure.

Bruxism has been shown to significantly affect not only implant failure [21], implant fracture [13], and marginal bone loss negatively [22], but also an increased prevalence of prosthesis failure and technical complications in implant-supported restorations in comparison to patients not presenting bruxism [14,18]. The condition is suggested to generate an overload of prosthetic rehabilitations on implants [23], as well as to be a risk factor for fractures of ceramics [24] and, in general, for the frequent need for technical interventions on implant-supported restorations [14,25,26,27].

Even though the difference in the distribution of bruxers between crowns placed in men and in women was not statistically significant, the higher risk of crown failure in men could be related to men having a higher maximal bite force than women [28], which is associated with greater muscle mass and size usually being observed in men [29]. Even though not all men were diagnosed as probable bruxers, higher bite force among male patients may predispose them not only to a higher risk of technical complications [30,31], but also to a higher implant failure rate [32].

A CSR of 92.2% was obtained for all single crowns after between 6 and 11 years, which is in line with the results of other studies reporting 10-year CSR, such as 91.7% [11], 95.2% [33], and 95.9% [34]. Small variations in the CSR may vary due to the distinct definitions for what failure would stand for, and/or for what kind of technical complications would be included as criteria for crown failure in different studies.

The most prevalent technical complication was mobility of the crown. The precise reason for the cases in the present study was unclear, but screw loosening is the most common problem, which is a greater risk to occur in single implant-supported crowns with external connection [35]. Reasons for screw loosening can be many and include inadequate pre-load, the anti-rotational characteristics of the implant-to-abutment interface, lack of precise fit of the mating components [36], tension on abutment resulting from this ill-fitting [37], less than ideal implant position, and inappropriate occlusal scheme or crown anatomy [38]. There is a recommendation to tighten the screw for an additional time in a second clinical appointment after the prosthesis is first screwed in place, in order to reduce the risk of screw loosening. This could be one of the possible reasons, but it was not possible to verify if this recommendation was followed by all dentists involved in the rehabilitations, unfortunately. The great advantage of screw-retained single crowns, which comprised the great majority of the cases in this study, is their possibility of easy screw re-tightening, retrievability, and repair [39].

Chipping of ceramic material was the second more prevalent technical complication. This can be associated with the nearly 94% of the cases in the present study being screw-retained crowns. The presence of unsupported ceramic around the prosthetic screw access hole in such crowns may decrease the fracture resistance [39,40]. An interesting finding was the occurrence of chipping among monolithic zirconia crowns. Veneered zirconia prostheses are more prone to chip-off fractures than monolithic zirconia prostheses, which do not have a veneering ceramic, and are expected to have less chipping and fracture complications [41]. Yet, chipping in monolithic zirconia can occur, as observed here as well as in other studies [42,43,44,45,46,47]. One of the possible explanations for this would be the so-called low-temperature degradation (LTD). This happens when the metastable tetragonal phase in stabilized monolithic zirconia transforms into a monoclinic phase in a humid environment [48,49,50], which starts at the surface and propagates into the material. Chewing forces can induce phase transformation around micro-cracks in the surface, leading to chipping of the outer surface [51]. Moreover, the results of an in vitro study suggest that variations in the implant-restoration angulation of one-piece screw-retained hybrid monolithic zirconia restorations with 15° may significantly affect their resistance to fractures [52]. Unfortunately, it was not possible to verify these factors in the present study, due to the retrospective nature of it. Cases of ceramic chipping were more prevalent among bruxers than in non-bruxers. This can be connected to the already aforementioned possible harmful effects of bruxism on implants and implant-supported restorations.

The limitations of the present study include its retrospective nature, which inherently results in flaws, manifested by gaps in information and incomplete records. Moreover, since this was not a prospective study, treatment was not standardized. Several professionals were involved in the treatment of these patients for the long time of observation of the study, and there must have been some variability of surgical and prosthetic approaches and dental laboratory techniques applied by these different professionals.

## 5. Conclusions

Probable bruxism and patient’s sex were factors associated with a higher risk of failure of implant-supported single crowns. The most commonly observed technical complications were mobility of the crown and chipping of the ceramic material.

## Figures and Tables

**Table 1:** Descriptive data of the implant-supported single crowns included in the study, with follow-up time between the different factors. The statistical unit is the single crown, not the patient.

Factor	Number of Crowns (%)	Failure (*n*)	Follow-Up (Months) Mean ± SD (Min–Max)
Sex			
Men	145 (40.5)	12	56.1 ± 31.5 (0.2–146.2)
Women	213 (59.5)	8	56.7 ± 28.5 (0.7–139.0
Age			
<60 years	240 (67.0)	15	53.5 ± 29.9 (0.7–139.0)
≥60 years	118 (33.0)	5	62.6 ± 28.3 (0.2–146.2)
Jaw			
Maxilla	166 (46.4)	9	52.0 ± 31.0 (0.2–146.2)
Mandible	192 (53.6)	11	60.4 ± 28.0 (1.4–139.0)
Prosthesis region			
Anterior	35 (9.8)	0	50.4 ± 35.5 (4.4–137.2)
Posterior	323 (90.2)	20	57.2 ± 29.0 (0.2–146.2)
Tooth type			
Incisor	27 (7.5)	0	50.0 ± 36.7 (4.4–137.2)
Canine	8 (2.2)	0	51.8 ± 33.5 (13.1–119.2)
Premolar	123 (34.4)	9	54.9 ± 33.3 (0.2–146.2)
Molar	200 (55.9)	11	58.6 ± 26.0 (1.4–137.5)
Crown fixation			
Cemented	23 (6.4)	0	65.6 ± 40.7 (1.8–137.2)
Screwed	335 (93.6)	20	55.9 ± 28.8 (0.2–146.2)
Crown material			
Metal–ceramic (CoCr)	111 (31.0)	7	52.6 ± 28.6 (0.2–137.5)
Metal–ceramic (Titanium)	165 (46.1)	12	63.3 ± 29.0 (0.7–146.2)
Metal–ceramic (Gold)	12 (3.3)	0	51.1 ± 37.3 (7.8–137.2)
Full ceramic	70 (19.6)	1	47.6 ± 28.6 (1.4–124.7)
Porcelain veneer	2 (2.9)	0	36.7 ± 4.7 (33.4–40.0)
Porcelain monolithic	2 (2.9)	0	42.5 ± 24.3 (25.4–59.7)
Zirconia veneer	22 (31.4)	1	43.7 ± 30.1 (4.4–119.2)
Zirconia monolithic	39 (55.7)	0	50.1 ± 30.1 (1.4–124.7)
Unknown	5 (7.1)	0	51.4 ± 19.5 (22.7–73.8)
Crown occluding to			
Natural teeth	104 (29.0)	7	49.8 ± 33.1 (1.4–137.5)
Teeth with filling	143 (39.9)	6	56.1 ± 26.5 (0.2–139.0)
Tooth-supported (metal–)ceramic prosthesis	78 (21.8)	6	62.0 ± 31.0 (1.4–146.2)
Implant-supported (metal–)ceramic prosthesis	25 (7.0)	1	67.2 ± 23.5 (35.8–110.0)
Metal–acrylic prosthesis	1 (0.3)	0	43.7
Nothing	7 (2.0)	0	66.0 ± 25.8 (28.5–97.5)
Treatment provider			
Dental student	236 (65.9)	15	60.2 ± 29.7 (1.8–146.2)
Dentist	122 (34.1)	5	49.4 ± 28.5 (0.2–137.2)
Probable bruxism			
No	257 (71.8)	9	54.7 ± 29.8 (0.2–146.2)
Yes	101 (28.2)	11	61.2 ± 29.0 (1.8–139.0)
Smoking			
No	282 (78.8)	19	57.8 ± 30.0 (0.2–146.2)
Yes	76 (21.2)	1	51.6 ± 28.2 (2.5–118.9)

**Table 2:** Life-table survival analysis showing the cumulative survival rate of implant-supported single crowns.

Interval Start Time (Years)	Number Entering Interval	Number Withdrawing during Interval	Number Exposed to Risk	Prosthesis Failure	Survival Rate within Each Interval—ISR (%)	Cumulative Proportion Surviving at End of Interval—CSR (%)	Standard Error (%)
0	358	31	342.5	9	97.4	97.4	0.9
1	318	26	305.0	5	98.4	95.8	1.1
2	287	23	275.5	0	100.0	95.8	1.1
3	264	58	235.0	3	98.7	94.6	1.3
4	203	48	179.0	2	98.9	93.5	1.5
5	153	57	124.5	0	100.0	93.5	1.5
6	96	43	74.5	1	98.7	92.2	1.9
7	52	26	39.0	0	100.0	92.2	1.9
8	26	14	19.0	0	100.0	92.2	1.9
9	12	8	8.0	0	100.0	92.2	1.9
10	4	1	3.5	0	100.0	92.2	1.9
11	3	3	1.5	0	100.0	92.2	1.9

**Table 3:** Univariate Cox proportional hazard models for implant-supported single crown failure.

Factor	Hazard Ratio (95% CI)	*p* Value
Sex		
Men	1	
Women	0.425 (0.174, 1.040)	0.061
Age		
<60 years	1	
≥60 years	0.615 (0.223, 1.697)	0.348
Jaw		
Maxilla	1	
Mandible	0.948 (0.392, 2.289)	0.905
Prosthesis region		
Anterior	1	
Posterior	23.326 (0.030, 18201)	0.354
Tooth type		
Incisor	^a^	
Canine	^a^	
Premolar	1	
Molar	0.678 (0.281, 1.639)	0.388
Crown fixation		
Cemented	1	
Screwed	22.587 (0.011, 46390)	0.423
Crown material		
Metal–ceramic (CoCr)	1	
Metal–ceramic (Titanium)	1.049 (0.412, 2.673)	0.920
Full ceramic	0.234 (0.029, 1.904)	0.175
Metal–ceramic (Gold)	^a^	
Crown occluding to		
Natural teeth (restored or not)	1	
Tooth-supported fixed prosthesis	1.387 (0.527, 3.653)	0.508
Implant-supported fixed prosthesis	0.648 (0.085, 4.957)	0.676
Treatment provider		
Dental student	1	
Dentist	0.713 (0.258, 1.967)	0.513
Probable bruxism		
No	1	
Yes	2.962 (1.227, 7.152)	0.016
Smoking		
No	1	
Yes	0.196 (0.026, 1.462)	0.112

^a^ No events of crown failure. 95% CI—95% confidence interval.

**Table 4:** Multivariate Cox proportional hazard model for implant-supported single crown failure.

Factor	Hazard Ratio (95% CI)	*p* Value
Sex		
Men	1	
Women	0.384 (0.156, 0.943)	0.037
Probable bruxism		
No	1	
Yes	3.226 (1.332, 7.814)	0.009

95% CI—95% confidence interval.

**Table 5:** Occurrence of technical complications.

Technical Complication	Number of Events (Number of Crowns)
Mobility of the crown	74 (49) 1×: 34 crowns, 2×: 9 crowns, 3×: 4 crowns, 5×: 2 crowns
Ceramic chipping	46 (42 ^a^) 2×: 4 crowns
Loss of screw hole sealing	41 (36) 2×: 5 crowns
Large fracture of ceramic material	10 (10 ^b^)
Prosthetic abutment fracture	2 (2)
Crown decementation	1 (1)
Prosthetic screw fracture	0 (0)

^a^ Crown material of the 42 cases (number of cases/total number of crowns): metal–ceramic titanium (24/165), metal–ceramic CoCr (13/111), zirconia monolithic (4/39), full ceramic of unknown specific material (1/5). ^b^ Crown material of the 10 cases (number of cases/total number of crowns): metal–ceramic titanium (5/165), metal–ceramic CoCr (3/111), zirconia veneer (1/22), full ceramic of unknown specific material (1/5).

## Data Availability

Restrictions apply to the availability of these data. Data were obtained from patients treated at the Faculty of Odontology, Malmö University, Malmö, Sweden, and cannot be shared, in accordance with the General Data Protection Regulation (EU) 2016/679.
